# The unexpected importance of the fifth digit during stone tool production

**DOI:** 10.1038/s41598-019-53332-w

**Published:** 2019-11-13

**Authors:** Alastair J. M. Key, Christopher J. Dunmore, Mary W. Marzke

**Affiliations:** 1 School of Anthropology and Conservation, University of Kent, Canterbury, Kent UK; 2 School of Human Evolution and Social Change, Arizona State University, Tempe, AZ USA

**Keywords:** Archaeology, Biological anthropology

## Abstract

Unique anatomical features of the human hand facilitate our ability to proficiently and forcefully perform precision grips and in-hand manipulation of objects. Extensive research has been conducted into the role of digits one to three during these manual behaviours, and the origin of the highly derived first digit anatomy that facilitates these capabilities. Stone tool production has long been thought a key influence in this regard. Despite previous research stressing the unique derived morphology of the human fifth digit little work has investigated why humans alone display these features. Here we examine the recruitment frequency, loading magnitude, and loading distribution of all digits on the non-dominant hand of skilled flintknappers during four technologically distinct types of Lower Palaeolithic stone tool production. Our data reveal the fifth digit to be heavily and frequently recruited during all studied behaviours. It occasionally incurred pressures, and was used in frequencies, greater or equal to those of the thumb, and frequently the same or greater than those of the index finger. The fifth digit therefore appears key to >2 million years of stone tool production activities, a behaviour that likely contributed to the derived anatomy observed in the modern human fifth ray.

## Introduction

Forceful precision grips and proficient in-hand manipulation underpin many of the behaviours considered unique to modern humans and our hominin ancestors^1–4^. While the ability of other primates to manipulate objects with force and/or precision is increasingly being recognised^5–9^, available evidence still indicates that modern humans more frequently or easily generate greater forces and dexterity during these behaviours^10,11^. Due to the human thumb’s robust skeletal morphology^12,13^, unique muscular anatomy^14^ and representation as a “defining” human feature^15^, it has been the focus of research into the evolution of hominin manual capabilities^16–21^. This has often been to the exclusion of the other four digits, in particular the fourth and fifth, whose derived traits are not as well explored and explained.

Digits two and three have been investigated in multiple important works, partly due to their frequent employment in grips used to wield hammerstones and flaked stone tools^2,22,23^. To date, analysis of digits four and five is often, but not always^24^, limited to examining their length relative to the first digit^17,25^.

The human fifth digit is relatively independent, unlike the fourth which during flexion and extension can also unintentionally recruit the third and fifth^26,27^. This independence is in part due to the saddle- shaped articulation for the fifth metacarpal base on the hamate, which allows the fifth metacarpal to flex and rotate at the same time, and better oppose the thumb and the rest of the hand^4,28^. This morphology also provides for a greater range of movement than in the other non-pollical metacarpals^29,30^. Movement of the fifth carpometacarpal (CMc) joint in this manner allows for a greater degree of curvature in the transverse metacarpal arch of the human palm relative to other apes^29,31^. In other great apes the fifth CMc joint is not sellar, and the hook of the hamate buttresses the metacarpal, limiting abduction from the midline of the hand as well as axial rotation, movements that permit opposition of the fifth digit with the first^28^. Combined with ulnar expansion of the palmar aspect of the fifth metacarpal head the unique morphology of humans at this joint, allows for effective opposition of the fifth digit to the thumb^28^.

Opposition of the fifth finger to the rest of the hand, or a grasped object, is crucial to in-hand manipulation, the forceful cradle precision grip required to produce and use flaked stone tools^2,32^, and to manipulating cylindrical objects in a power grip^33^. The human fifth metacarpal is also unique in being second only to the first metacarpal in robusticity^33^, reflecting its ability to sustain large loads. In addition, a site-specific concentration of trabecular bone volume has been found beneath the opponens digiti minimi muscle insertion site, along the ulno-palmar aspect of the fifth metacarpal shaft in human hands, suggesting frequent recruitment of this muscle, likely during the opposition of the fifth finger to the thumb^34^.

**Figure 1:**
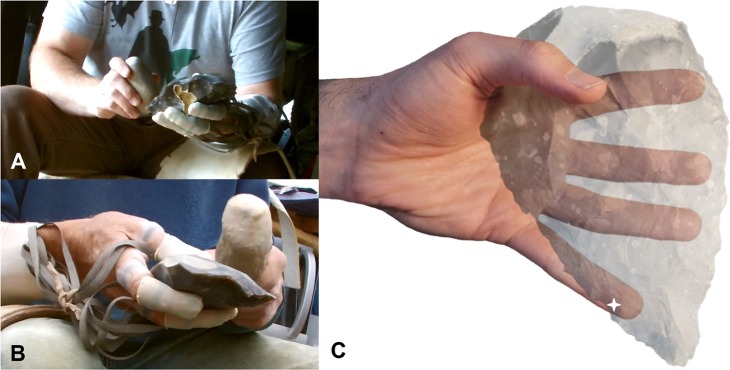
Knappers five (**A**) and four (**B**) during their LAH reduction sequence. The video stills are taken prior to flakes being removed with hard hammer and soft hammer percussors. Although not visible in the video stills, note the fifth digit’s location beneath the point of impact (indicated with a star) (**C**) (see also: Supplementary Information Video 1).

Experimental research during the 1980s and 90s provided insight into the evolution of these uniquely human traits. Marzke and Shackley^22^ highlighted that the fifth digit was frequently recruited to control stone cores during flake removals and to adjust the flake platform’s angle relative to the hammerstone. Further, the fourth and fifth digits were demonstrated to aid the stabilisation and securing of stone tools and antler billets during their use. More recent studies emphasised the variable, but at times vital, stabilising and manipulative role of the fifth digit during stone tool production and use^19,23,32^. Other studies of stone tool related activities^24^ and carrying^35^ have identified more limited roles for the fourth and fifth digits when compared to the radial digits. However, electromyographic studies of muscles serving the fifth digit (flexor and abductor digiti minimi muscles) during stone tool production revealed their recruitment during hammerstone strikes^32^. These muscles were particularly heavily recruited on the non-dominant, core holding hand, where the fifth digit was argued to play a critical role in stabilizing the core against hammerstone impact forces^32^ (Fig. 1). Since the early studies by Marzke and colleagues^22,32^, there has been little research specifically concerning the evolutionary history of the hominin fifth digit, its unique anatomy^36,37^, or its role in gripping and in-hand manipulation during stone tool related behaviours^38^. In turn, our understanding of the evolution of ulnar (digits 4–5) hand morphology in fossil hominins and modern humans is highly limited, particularly when compared to the thumb.

Here, we approach the question of why modern humans display derived fifth ray anatomy and increased joint mobility from an experimental perspective. We examine the pressure levels experienced by nine expert knappers across their non-dominant hand during four technologically distinct Lower Palaeolithic stone tool production behaviours (Fig. 1). Through analyses of pressure distribution, magnitude, and loading frequencies, we investigate the role of the fifth digit during stone tool production, the biomechanical stress it experiences, and how this relates to the type of stone tool being produced. Further, we compare these data directly with the other non-pollical digits to explore the comparative evolutionary history of the radial and ulnar sides of the hominin hand within a Lower Palaeolithic behavioural context.

## Results

### Pressure distribution

**Figure 2:**
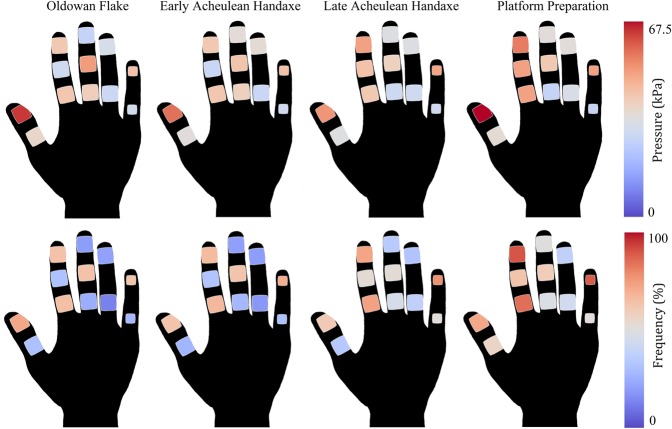
Colour maps detailing the distribution of mean pressure data (top row) and frequency of recruitment data (bottom row) during the four stone tool production behaviours.

Post-hoc Dunn’s tests between the twelve sensors during the four reduction types are presented in the accompanying Supplementary Information Tables 2–5. These statistical comparisons highlighted significant differences in pressure between sensors distributed across the hand during all reduction behaviours (Figs 1 and 2). On almost all occasions, the sensor on the distal phalanx of the thumb (DP1) experienced significantly greater relative pressure than all other sensors during the four tool production strategies (*p* = <0.0001). The only two exceptions were during the late Acheulean handaxe (LAH) sequence, where the sensors on the distal phalanx of the second (DP2) and fifth (DP5) digit, recorded no significant pressure differences relative to DP1 (Supplementary Information Table 4).

DP2 and DP5 sensors are the next most heavily recruited across all four behaviours (Supplementary Information Tables 2–5). For both sensors the LAH and platform preparation (PP) data returned the greatest number of significant differences, each displaying substantially greater pressure than nine and seven other sensors, respectively. The other two reductions (Oldowan flake [OF] and early Acheulean handaxe [EAH]) returned six significant differences for these two sensors, thus still indicating their heavy recruitment. No significant differences were observed between DP2 and DP5 across all four comparisons, indicating their similar pressure levels.

Results are less consistent across the four behaviours for the remaining sensors (Supplementary Information Tables 2–5). Interestingly, sensors on the intermediate phalanges of digits two and three (IP2, IP3) and the proximal phalanx of digit two (PP2) also experienced heavy loading, returning between three and six significantly greater results relative to the other sensors, in each of the four reduction types. Although relative to DP1, DP2 and DP5 these values were usually reduced. In comparison, the distal phalanx of digits three and four were not heavily loaded during the reduction behaviours. Nor were the proximal phalanges of digits one, three, four or five.

### Pressure magnitude

Maximum recorded pressures are considerable, frequently ranging between 150–250 kPa (Supplementary Information Table 1). Across all extracted peak values, however, pressures exceeding 100 kPa were relatively rare (Supplementary Information Fig. 1; Supplementary Information Tables 6–9). Typically, 30–50% of pressure values for all sensors in all reductions ranged between 20–40 kPa (Supplementary Tables 12–15.

Beyond these values there are differences between sensors and reduction types. Consistent with the pressure distribution results, DP1, DP2, DP5, IP3, PP2, and PP3 returned pressure values of 40–60 kPa 20–30% of the time, while DP3, DP4, IP2, PP1, PP4, and PP5 displayed these values < 20% of the time (often <10%). The sensors that frequently (30–50%) returned values < 20 kPa were most often proximally located (PP1, PP3, PP4 and PP5), but also include IP2 and DP3. Only DP1, DP2, and DP5 repeatedly returned >20% of their values as being 60 kPa or greater across all four reductions.

**Figure 3:**
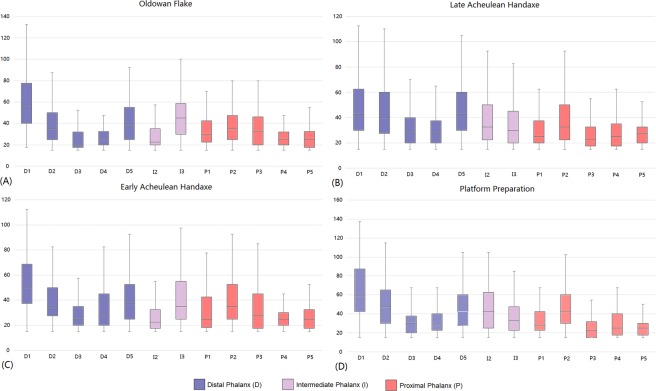
Boxplots displaying pressures experienced across the twelve sensors examined here. Data from the Oldowan (**A**), Early Acheulean Handaxe (**B**), Late Acheulean Handaxe (**C**) and Platform Preparation (**D**) reduction behaviours are depicted. Blue plots correspond to sensors on distal phalanges, while pink and red are sensors on intermediate and proximal phalanges, respectively. ‘D’, ‘I’ and ‘P’ correspond to the distal phalanx, intermediate phalanx and proximal phalanx sensors, respectively. Sensors numbers correspond to the respective digit.

Pressures above 100 kPa were rare. In total, there were 89, 112, 255 and 90 values > 100 kPa during the OF, EAH, LAH and PP behaviours, respectively. Differences between reduction types were not, however, proportionate to the number of mass removal events in each. Indeed, proportionately, PP behaviours more frequently recorded values >100 kPa. Of these, the majority of values were recorded on DP1 during the OF, EAH and PP behaviours, while DP1, DP2 and DP5 all displayed ~50 of these values during the LAH reduction. Mean peak pressure values accord with the range data already described, with all sensors ranging between 25–65 kPa, dependent on the type of reduction undertaken and the position of the sensor on the hand (Fig. 3).

### Recruitment frequency

Recruitment frequency data identify DP1, DP2, PP2, IP3, and DP5 as the five most recurrently loaded sensors (Table 1). They experience loading during 60–90% of all analysed manual behaviours in all tool production types. The remaining sensors display recruitment frequencies ranging between 13–63%, with the proximal phalanges of digits one, three, four and five typically being the lowest. In three out of four behaviours, DP5 is the most frequently loaded sensor. The only exception is the Oldowan reduction, where the thumb is recruited most often. It is notable that all sensors, bar DP1 and IP3, display increased recruitment frequencies during the LAH and PP behaviours, relative to the Oldowan and EAH sequences. This is particularly clear for DP2 and DP5, where recruitment frequencies are as high as 90% and 88% during the PP behaviours, respectively (Table 1).

**Table 1:** Digit recruitment frequencies expressed as a percentage relative to the total number of manual behaviours recorded.

		Sensor											
		PD1	PP1	PD2	IP2	PP2	PD3	IP3	PP3	PD4	PP4	PD5	PP5
OF (n = 523)	Instances	377	160	334	169	340	102	335	131	115	70	331	145
	Frequency (%)	78	31	64	32	65	20	64	25	22	13	63	28
EAH (n = 789)	Instances	499	214	524	263	532	168	496	219	154	151	555	242
	Frequency (%)	69	27	66	33	67	21	63	28	20	19	70	31
LAH (n = 1251)	Instances	761	432	909	666	920	438	669	582	413	481	962	644
	Frequency (%)	63	35	73	53	74	35	53	47	33	38	77	51
PP (n = 271)	Instances	194	155	242	171	229	137	162	130	104	121	238	140
	Frequency (%)	75	57	90	63	85	51	60	48	39	45	88	52

## Discussion

We have recorded the distribution, magnitude, and frequency of pressures acting on the non-dominant hand of skilled flintknappers during multiple stone tool production procedures. Our results reveal a unique radio-ulnar loading pattern observed in no other Plio-Pleistocene manual behaviours analysed to date. The uniqueness of this pattern lies in the heavy recruitment of the fifth finger, alongside that of the thumb and index finger. These data help explain derived manual anatomy in fossil hominins and modern humans.

Our data indicate substantial loading on distal aspects of the thumb, second and fifth digits, and although more limited, also proximally on the second and third digits. Other phalanges returned lower loading levels. This distribution is consistent across records of mean and maximum pressure magnitude and loading frequency (Fig. 2).

Previous studies of manual loading during Plio-Pleistocene activities have either not investigated the fifth digit^18,19,39,40^, or identified reduced loading relative to digits one-to-three^24,35^. Our results uniquely demonstrate the fifth digit to be heavily and frequently loaded during stone tool production; being equal second in terms of loading magnitude, and in three out of four reduction types, the most frequently recruited. It therefore appears that the fifth digit could have experienced selective pressure in response to these high demands, subsequent to the onset of habitual freehand stone tool production after ~2.6 Mya.

The significantly greater pressures experienced by the thumb, relative to digits two-to-five, during the OF, EAH and PP behavioural sequences is consistent with previous experimental research examining loading during stone tool production in both the non-dominant and dominant hand^19,24^. Combined, these three studies support the hypothesis that the production of flaked stone tools likely contributed to the robust first digit skeletal and musculature anatomy observed in the hominin lineage^3,4^.

High loading on the fifth digit is not necessarily surprising given previous grip analyses and electromyographic studies of stone tool production^19,22,32^. Nevertheless, it is surprising that the magnitude and frequency of this loading is greater or equal to that of the thumb in some instances and similar to that of the second digit. As previously detailed^19,22,32^, the fifth digit is often located directly beneath or very close to the point of impact when flakes are removed via freehand percussion (Fig. 1). This allows flakes to be supported during removals and their platforms to be directed towards the knapper’s body and, importantly, their dominant hand; in turn, allowing percussors to accurately and repeatedly strike flake platforms using free-flowing and comfortable arching motions by the dominant arm^41^. As percussors strike a core supported in this way a proportion of the impact force propagates through the stone and removes the flake. This force will disperse through all manual elements in contact with the core proportionate to hammerstone strike direction, core morphology and manual element distribution across the core’s surface. Understandably, the fifth digit experiences a high proportion of this force due to its positioning in line with the point of impact (Fig. 1). High-speed videos (800 f/s) in Supplementary Information Video 1 highlight the dispersion of force through the fifth digit. It is evident that during both hard hammer and soft hammer flake detachments the fifth digit is directly beneath the point of impact, with reaction forces extending the digit dorsally. To prevent hyperextension and the loss of the flake by not being supported, it is essential for the fifth digit to resist these forces, in turn resulting in the high pressures recorded here. Additionally, the essential role of the thumb in securing the core into to the hand can also be observed.

High loading on the distal fifth digit, particularly during platform preparation events and when securing the core against the leg, may also result from its ability to oppose the thumb when creating a secure inclusive grip around a core^3^. Certainly, as the most ulnarly located digit, when abducted it extends the reach of the hand and can directly oppose the thumb across the midpoint of the palm. Increased pressure values on the fifth digit during the production of late Acheulean handaxes (LAH, PP), in particular, can be attributed to their low thickness to width ratios, smaller size, increased shaping, and greater requirement to remove long, elongated ‘thinning’ flakes^38,42,43^.

The magnitude of loads experienced by digits on the non-dominant hand have been demonstrated to be considerable. Indeed, the pressure ranges recorded here are broadly equal to those observed on the dominant hand of knappers^18,24,39^, a note of importance given recent statements to the contrary. The upper limits of 150–250 kPa detailed here are consistent with pressures recorded from the dominant hand of the same individuals^39^ and non-skilled individuals^24^ during flake tool production.

There do, however, appear to be differences in the frequency with which high loads are experienced on individual phalanges (sensors) between the non-dominant and dominant hand. Certainly, here pressures above ~100 kPa are infrequent and account for a low proportion of data values (Supplementary Information Tables 6–9). In part, this may reflect differences in experimental design as all mass removal events, no matter how small, were recorded in the present study. Additionally, previous dominant hand research has typically utilized stop-and-start knapping sequences that are less typical of natural knapping behaviours^18,24,39^. We do, however, also consider this to reflect important recruitment pattern differences between the dominant and non-dominant hand during stone tool production. Certainly, in addition to their non-dominant hand knappers frequently support cores using their leg and palm, consequently providing an additional surface to distribute forces. Moreover, cores typically have larger surface areas that, as identified here, more frequently recruit ulnarly located digits and proximally located phalanges during their manipulation. Dominant hand pressure distribution appears to focus solely on digits one-to-three^24^. When compared at a whole hand level (i.e. cumulative peak pressures from 12 identically located sensors), however, the non-dominant hand experiences cumulative loading magnitudes roughly four times as large as the dominant hand^24,38^.

Loading frequencies on the non-dominant hand broadly mirror the distribution of variation in pressure magnitude, including the heavy recruitment of the fifth distal phalanx. It is, perhaps, not surprising that these metrics of manual recruitment are related, however these metrics provide details essential for understanding the derived traits observed in the human hand^24^. Our frequency data broadly corroborates previous findings that the first digit on the non-dominant hand is recruited more often than the second or third during stone tool production^19^.

The data presented here underlines the important role played by the fifth digit during stone tool production and helps explain the derived anatomy observed in the modern human fifth digit. It is interesting that many of distinctively human features of the fifth ray are first seen among current hominin fossils in *Homo naledi*^44^, dated to 236–325 ka^45^, and in Neandertals^36^. Its heavy and frequent recruitment, which is occasionally greater or equal to the thumb, and the same or greater than the index finger, hints at the complex array of recruitment patterns likely experienced by the hominin hand during the Plio-Pleistocene. To date, investigations into the derived anatomy of the modern human hand have, understandably, focused on lithic-related behaviours. However, numerous manual activities likely undertaken by early hominins, and perhaps essential to their survival and reproductive success, have not yet been examined (e.g. digging stick and spear use, manipulating food, grooming). While work has started to address this deficit^7,24,35,46^, further studies investigating such behaviours are necessary if we are to fully understand the evolutionary history of the hominin hand. Certainly, as highlighted here, the demands placed on early hominin hands were likely far more diverse than current evidence indicates.

## Material and Methods

### Stone tool production procedures

Pressure values were recorded from the non-dominant hand of participants using a wireless Novel Pliance® pressure system comprising of ten 17 × 17 mm sensors and two 10 × 10 mm sensors (Figs 1 and 3). Sensors were attached to the palmar side of the distal phalanges of digits one-five, the intermediate phalanges of digits two and three, and the proximal phalanges of digits one-five. The two 10 × 10 mm sensors were secured to the distal and proximal phalanges of the fifth digit. Double-sided tape, Velcro straps and finger cots secured each sensor. Sensors were ‘zeroed out’ prior to data collection and all pressure data were recorded at a rate of 50 Hz.

Nine experienced flintknappers took part in the study (all were capable of consistently producing late Lower Palaeolithic handaxes when required). Each was asked to undertake three stone tool production sequences; Oldowan flake and core production, early Acheulean handaxe, and late Acheulean handaxe production. Hard hammer percussion was used in all three sequences. Soft hammer percussion and platform preparation events were only used during the LAH reduction. All tool production events were recorded using a HD video camera. Knappers were allowed to produce tools at their own pace and using their own hammerstones and billets. Ethical approval was granted by the School of Anthropology and Conservation Ethics Committee (University of Kent; Ref. Ares 19065). All individuals gave informed consent and experiments were conducted in accordance with industry (AAPA) guidelines.

### Pressure data

Sensors collected data throughout each reduction sequence, however, pressure data extracted for analysis only refers to manual behaviours that removed mass from the core. Within Oldowan flake and EAH reductions this only referred to attempted flake detachments, while the LAH sequence also included PP events (see also^38^). Prior to starting a reduction knappers forcefully pinched between the thumb and index finger. This created a point of known manual pressure that was easily identifiable in both the pressure data and the video recording. Subsequently, behaviours observed in the video could be matched with their accompanying pressure output and it was possible to identify individual mass removal events within the pressure data stream.

Each sensor’s peak pressure during each mass removal event was extracted for analysis. During attempted flake detachments, peak pressures were extracted from two second intervals (one second prior to and after the point of impact). Platform preparation events were variable in duration and peak pressures were identified as the highest values recorded from the start to end of these behaviours. Digits are intermittently recruited when securing stone cores^19^, and in turn, pressure values of 0 kPa were at times recorded by sensors during mass removal events. To avoid examining loading as a combined function of peak pressures and the frequency of digit recruitment, pressure records of zero were excluded from analyses (other than those examining loading frequency). In turn, statistical comparisons of distribution and magnitude focus solely on peak pressure differences between digits when they are actively recruited to manipulate a core.

Data from all participants were combined for each of the statistical analyses performed. Participant seven’s distal sensor on the first digit broke during data collection. All analyses for this sensor are, therefore, derived from the remaining eight knappers and are adjusted accordingly. Supplementary Tables 1–15 reveal that mean, standard deviation, and range pressure values remain stable across the other 11 sensors with and without participant seven; hence the continued inclusion of their data in the other sensors.

### Statistical analysis

Previous investigations of manual loading during stone tool related behaviours have focused on pressure distribution^18^, magnitude^38,39^, magnitude and distribution^28^, force magnitude^40^ or force distribution and digit recruitment frequency^19^. Here, we conduct analyses of pressure distribution, magnitude and frequency. Each is analysed independently for the Oldowan flake, EAH and LAH reduction sequences. The LAH sequence was separated further into flake removals (LAH) and platform preparation events (PP), in turn creating four stone tool behaviours.

### Pressure distribution

To examine how loading varies across the non-dominant hand peak pressure records were compared between sensors. Shapiro-Wilk tests confirmed that all 48 sets of peak pressure data sets (twelve sensors across four reduction types) were not normally distributed (*p* = < 0.05). In turn, Kruskal-Wallis and post-hoc Dunn’s tests were used for these comparisons. Relative pressure measures were used to investigate loading distribution throughout the hand; absolute pressures could have led to erroneous results if knappers with particularly high or low values had individually favoured a particular finger. Relative data were calculated by dividing each knapper’s pressures by their average per knapping behaviour. Participant seven was not included in this analysis as the loss of one sensor would create non-comparable averages for the other sensors on this hand. Significance was assumed at p < 0.05 subsequent to a Bonferroni correction.

### Pressure magnitude

Beyond basic descriptive data for each sensor during each reduction type (Supplementary Information Table 1), magnitude of peak pressure was examined between sensors relative to the frequency that specific loading values were recruited. Specifically, the pressure range recorded during this experiment (0–252.5 kPa) were divided into thirteen 20 kPa segments (e.g. 0–20, 20.1–40, 40.1–60) and the percentage of peak pressure records for each sensor falling into these values were recorded.

### Recruitment frequency

Recruitment frequency was recorded as the percentage of times that a sensor experienced a load during mass removal events relative to the total number of flake detachments and platform preparation events that were undertaken during that reduction. For example, the total number of flake removals attempted across the nine participant’s Oldowan core reductions was 523. Of these flake removal events, the distal sensor on the thumb registered a load >0 kPa 377 times. In turn, this sensor had a recruitment frequency of 72%. This calculation was repeated for all twelve sensors in each of the four reduction behaviours.

## Supplementary information

Supplementary Video 1.

Supplementary Video 2.

Supplementary Information 1

Supporting data.

## Data Availability

The data supporting this article have been uploaded as electronic supplementary material.
