# Supplementary material for: The unexpected importance of the fifth digit during stone tool production
Source: Sci Rep. 2019 Nov 13;9:16724. doi: 10.1038/s41598-019-53332-w (PMC6853985; doi:10.1038/s41598-019-53332-w)
Supplement: Supplementary file 3 — Supplementary Information 1 [file 41598_2019_53332_MOESM3_ESM.pdf]

Supplementary Information File

The unexpected importance of the fifth digit during stone tool production

Alastair J. M. Key[1]*, Christopher J. Dunmore[1], Mary W. Marzke[2]

*Corresponding author: a.j.m.key@kent.ac.uk

[1]School of Anthropology and Conservation, University of Kent, Canterbury, Kent (UK)

[2] School of Human Evolution and Social Change, Arizona State University, Tempe, AZ (USA)

Supplementary Table 1: Descriptive data for the twelve sensors during the three types of stone tool reduction. Minimum values always equal 15 kPa, except for DP1 during the Oldowan reduction, which recorded a minimum value of 17.5 kPa. These data exclude values of '0' when sensors were not loaded.

		Sensor											
		**DP1**	**PP1**	**DP2**	**IP2**	**PP2**	**DP3**	**IP3**	**PP3**	**DP4**	**PP4**	**DP5**	**PP5**
**Oldowan Flake**	**Mean**	61.7	36.3	40.3	28.6	40.4	26.0	48.8	38.7	30.3	27.6	41.4	28.6
	**SD**	29.3	22.5	20.7	12.9	21.8	13.4	27.5	27.4	18.4	12.9	20.4	14.2
	**Max**	162.5	145	125	85	155	85	222.5	195	100	77.5	150	90
**Early Acheulean Handaxe**	**Mean**	54.9	33.5	39.9	27.1	40.7	34.0	41.1	38.3	34.7	27.0	41.6	28.6
	**SD**	26.3	22.2	20.0	13.2	24.1	26.3	23.7	29.4	20.7	12.3	22.8	14.0
	**Max**	172.5	197.5	145	77.5	252.5	162.5	192.5	170	110	87.5	162.5	87.5
**Late Acheulean Handaxe**	**Mean**	50.0	32.7	47.5	41.6	40.1	31.9	38.2	28.0	32.0	28.3	47.3	28.6
	**SD**	27.3	21.1	29.3	26.6	25.5	15.4	25.9	14.9	18.4	14.6	25.3	11.7
	**Max**	190	162.5	228.5	207.5	232.5	82.5	197.5	115	132.5	132.5	155	92.5
**LAH Platform Preparation**	**Mean**	67.5	34.2	53.2	47.8	47.5	33.8	40.0	25.9	33.6	30.9	47.8	25.7
	**SD**	34.7	18.7	31.2	27.3	24.3	22.5	27.4	13.6	18.0	17.3	25.1	9.0
	**Max**	187.5	130	232.5	147.5	142.5	222.5	197.5	87.5	97.5	115	145	57.5

Supplementary Table 2: Relative peak pressure comparisons between sensors during Oldowan flaking. In each case pressure values were divided by the average pressure across all sensors per knapper. Shapiro-Wilk tests confirm neither dataset was normally distributed. A Kruskal-Wallis and post-hoc Dunn's tests were considered significant (bold) at $p < .05$ subsequent to a Bonferroni Correction.

**Oldowan Flake**												
**Digit**		**1st**		**2nd**			**3rd**			**4th**		**5th**
	**Phalanx**	**DP**	**PP**	**DP**	**IP**	**PP**	**DP**	**IP**	**PP**	**DP**	**PP**	**DP**
**1st**	**PP**	**.000**										
**2nd**	**DP**	**.000**	.465									
	**IP**	**.000**	**.047**	**.000**								
	**PP**	**.000**	.280	1.000	**.000**							
**3rd**	**DP**	**.000**	**.009**	**.000**	1.000	**.000**						
	**IP**	**.000**	**.000**	**.001**	**.000**	**.002**	**.000**					
	**PP**	**.000**	1.000	1.000	**.001**	1.000	**.000**	**.009**				
**4th**	**DP**	**.000**	.300	**.000**	1.000	**.000**	1.000	**.000**	**.001**			
	**PP**	**.000**	.078	**.000**	1.000	**.000**	1.000	**.000**	**.003**	1.000		
**5th**	**DP**	**.000**	.315	1.000	**.000**	1.000	**.000**	**.002**	1.000	**.000**	**.000**	
	**PP**	**.000**	.190	**.000**	1.000	**.000**	1.000	**.000**	**.005**	1.000	1.000	**.000**

Supplementary Table 3: Relative peak pressure comparisons between sensors during Early Acheulean handaxe flaking. In each case pressure values were divided by the average pressure across all sensors per knapper. Shapiro-Wilk tests confirm neither dataset was normally distributed. A Kruskal-Wallis and post-hoc Dunn's tests were considered significant (bold) at $p < .05$ subsequent to a Bonferroni Correction.

**Early Acheulean Flake**												
**Digit**		**1st**		**2nd**			**3rd**			**4th**		**5th**
	**Phalanx**	**DP**	**PP**	**DP**	**IP**	**PP**	**DP**	**IP**	**PP**	**DP**	**PP**	**DP**
**1st**	**PP**	**.000**										
**2nd**	**DP**	**.000**	**.000**									
	**IP**	**.000**	**.004**	**.000**								
	**PP**	**.000**	**.000**	1.000	**.000**							
**3rd**	**DP**	**.000**	1.000	**.000**	**.006**	**.000**						
	**IP**	**.000**	**.000**	1.000	**.000**	1.000	**.001**					
	**PP**	**.000**	1.000	**.000**	**.002**	**.000**	1.000	**.001**				

4th	DP	**.000**	1.000	.070	**.000**	.184	1.000	.711	1.000			
	PP	**.000**	1.000	**.000**	1.000	**.000**	1.000	**.000**	1.000	.249		
5th	DP	**.000**	**.000**	1.000	**.000**	1.000	**.000**	1.000	**.000**	.209	**.000**	
	PP	**.000**	1.000	**.000**	1.000	**.000**	1.000	**.000**	1.000	**.027**	1.000	**.000**

Supplementary Table 4: Relative peak pressure comparisons between sensors during Late Acheulean handaxe flaking. In each case pressure values were divided by the average pressure across all sensors per knapper. Shapiro-Wilk tests confirm neither dataset was normally distributed. A Kruskal-Wallis and post-hoc Dunn's tests were considered significant (bold) at $p < .05$ subsequent to a Bonferroni Correction.

Late Acheulean Handaxe												
**Digit**		**1st**		**2nd**			**3rd**			**4th**		**5th**
	**Phalanx**	**DP**	**PP**	**DP**	**IP**	**PP**	**DP**	**IP**	**PP**	**DP**	**PP**	**DP**
**1st**	**PP**	**.000**										
**2nd**	**DP**	.139	**.000**									
	**IP**	**.000**	**.000**	**.000**								
	**PP**	**.000**	**.000**	**.000**	1.000							
**3rd**	**DP**	**.000**	1.000	**.000**	**.000**	**.000**						
	**IP**	**.000**	**.000**	**.000**	.985	.244	.134					
	**PP**	**.000**	.908	**.000**	**.000**	**.000**	**.001**	**.000**				
**4th**	**DP**	**.000**	1.000	**.000**	**.000**	**.000**	1.000	**.000**	.714			
	**PP**	**.000**	1.000	**.000**	**.000**	**.000**	**.012**	**.000**	1.000	1.000		
**5th**	**DP**	1.000	**.000**	1.000	**.000**	**.000**	**.000**	**.000**	**.000**	**.000**	**.000**	
	**PP**	**.000**	1.000	**.000**	**.000**	**.000**	1.000	**.000**	**.018**	1.000	.162	**.000**

Supplementary Table 5: Relative peak pressure comparisons between sensors during Late Acheulean platform preparation. In each case pressure values were divided by the average pressure across all sensors per knapper. Shapiro-Wilk tests confirm neither dataset was normally distributed. A Kruskal-Wallis and post-hoc Dunn's tests were considered significant (bold) at $p < .05$ subsequent to a Bonferroni Correction.

LAH Platform Preparation												
**Digit**		**1st**		**2nd**			**3rd**			**4th**		**5th**
	**Phalanx**	**DP**	**PP**	**DP**	**IP**	**PP**	**DP**	**IP**	**PP**	**DP**	**PP**	**DP**
**1st**	**PP**	**.000**										
**2nd**	**DP**	**.000**	**.000**									
	**IP**	**.000**	**.000**	.053								
	**PP**	**.000**	**.000**	1.000	1.000							
**3rd**	**DP**	**.000**	1.000	**.000**	**.000**	**.000**						
	**IP**	**.000**	1.000	**.000**	**.014**	**.000**	1.000					
	**PP**	**.000**	**.012**	**.000**	**.000**	**.000**	**.014**	**.000**				
**4th**	**DP**	**.000**	1.000	**.000**	**.000**	**.000**	1.000	1.000	**.045**			
	**PP**	**.000**	1.000	**.000**	**.000**	**.000**	1.000	.428	.554	1.000		
**5th**	**DP**	**.000**	**.000**	1.000	1.000	1.000	**.000**	**.000**	**.000**	**.000**	**.000**	
	**PP**	**.000**	.301	**.000**	**.000**	**.000**	.319	**.001**	1.000	.983	1.000	**.000**

Supplementary Table 6: Pressure magnitude frequencies, expressed as a percentage, experienced during the Oldowan reduction sequence.

	**Pressure range (kPa)**	**Sensor (%)**											
		**DP1**	**PP1**	**DP2**	**IP2**	**PP2**	**DP3**	**IP3**	**PP3**	**DP4**	**PP4**	**DP5**	**PP5**
**Oldowan Flake**	**0-20**	1.6	23.1	18.9	33.7	12.1	56.9	11.9	26.7	33.0	32.9	13.9	37.9
	**20.1 - 40**	29.2	48.8	41.6	49.7	50.3	32.4	31.9	38.2	53.9	54.3	41.7	45.5
	**40.1 - 60**	27.3	18.8	22.8	14.8	23.5	8.8	34.3	24.4	5.2	8.6	29.3	13.1
	**60.1 - 80**	20.7	4.4	12.3	1.2	7.9	1.0	12.5	6.1	2.6	4.3	12.1	2.1
	**80.1 - 100**	10.1	1.3	3.3	0.6	4.1	1.0	4.5	1.5	5.2	0	2.1	1.4
	**100.1 - 120**	4.8	3.1	0.9	0	1.2	0	2.1	0.8	0	0	0.6	0
	**120.1 - 140**	5.0	0	0.3	0	0.6	0	1.5	0	0	0	0	0
	**140.1 - 160**	1.1	0.6	0	0	0.3	0	0.9	1.5	0	0	0.3	0

	**160.1 - 180**	0.3	0	0	0	0	0	0	0	0	0	0	0
	**180.1 - 200**	0	0	0	0	0	0	0	0.8	0	0	0	0
	**200.1 - 220**	0	0	0	0	0	0	0	0	0	0	0	0
	**220.1 - 240**	0	0	0	0	0	0	0.3	0	0	0	0	0
	**240.1 - 260**	0	0	0	0	0	0	0	0	0	0	0	0

Supplementary Table 7: Pressure magnitude frequencies, expressed as a percentage, experienced during the Early Acheulean Handaxe reduction sequence.

	**Pressure range (kPa)**	**Sensor (%)**											
		**DP1**	**PP1**	**DP2**	**IP2**	**PP2**	**DP3**	**IP3**	**PP3**	**DP4**	**PP4**	**DP5**	**PP5**
**Early Acheulean Handaxe**	**0-20**	1.6	33.2	13.0	38.4	16.4	41.1	18.1	34.2	27.3	31.8	16.0	36.4
	**20.1 - 40**	36.7	37.9	49.2	50.6	42.9	36.3	44.2	34.2	44.8	60.9	41.8	47.9
	**40.1 - 60**	30.7	20.6	26.5	5.7	23.9	11.9	20.6	16.9	18.2	3.3	27.2	12.0
	**60.1 - 80**	15.4	6.1	7.3	5.3	11.3	4.2	10.9	5.5	5.2	2.6	7.9	2.5
	**80.1 - 100**	9.0	1.4	2.1	0	3.0	2.4	4.4	4.1	2.6	1.3	4.5	1.2
	**100.1 - 120**	4.0	0	1.5	0	2.1	1.2	1.0	1.4	1.9	0	2.2	0
	**120.1 - 140**	1.6	0	0.2	0	0.4	1.8	0.2	1.8	0	0	0.2	0
	**140.1 - 160**	0.8	0	0.2	0	0	0.6	0.4	1.4	0	0	0	0
	**160.1 - 180**	0.2	0.5	0	0	0	0.6	0	0.5	0	0	0.2	0
	**180.1 - 200**	0	0.5	0	0	0	0	0.2	0	0	0	0	0
	**200.1 - 220**	0	0	0	0	0	0	0	0	0	0	0	0
	**220.1 - 240**	0	0	0	0	0	0	0	0	0	0	0	0
	**240.1 - 260**	0	0	0	0	0.2	0	0	0	0	0	0	0

Supplementary Table 8: Pressure magnitude frequencies, expressed as a percentage, experienced during the Late Acheulean Handaxe reduction sequence.

	**Pressure range (kPa)**	**Sensor (%)**											
		**DP1**	**PP1**	**DP2**	**IP2**	**PP2**	**DP3**	**IP3**	**PP3**	**DP4**	**PP4**	**DP5**	**PP5**
**Late Acheulean Handaxe**	**0-20**	5.5	31.9	11.3	16.5	20.1	29.7	25.1	38.1	27.4	38.5	10.1	28.7
	**20.1 - 40**	41.3	46.5	41.6	48.3	41.6	49.8	43.0	48.1	51.3	47.6	39.0	59.3
	**40.1 - 60**	28.0	11.8	22.2	18.3	23.0	12.6	19.4	9.6	14.3	10.2	29.1	9.6
	**60.1 - 80**	13.8	6.0	12.7	8.1	7.4	7.5	6.6	2.6	3.9	3.1	10.7	2.2
	**80.1 - 100**	5.5	2.1	7.3	3.9	4.3	0.5	2.7	1.2	1.7	0.2	6.7	0.2
	**100.1 - 120**	3.7	0.9	2.0	2.9	1.7	0	1.2	0.3	1.0	0.2	2.8	0
	**120.1 - 140**	0.9	0	1.4	1.1	0.2	0	0.4	0	0.5	0.2	1.4	0
	**140.1 - 160**	0.9	0.5	0.7	0.8	0.3	0	0.9	0	0	0	0.3	0
	**160.1 - 180**	0.3	0.2	0.4	0	0.3	0	0.4	0	0	0	0	0
	**180.1 - 200**	0.1	0	0.3	0	0	0	0.1	0	0	0	0	0
	**200.1 - 220**	0	0	0	0.2	0.1	0	0	0	0	0	0	0
	**220.1 - 240**	0	0	0.1	0	0.1	0	0	0	0	0	0	0
	**240.1 - 260**	0	0	0	0	0	0	0	0	0	0	0	0

Supplementary Table 9: Pressure magnitude frequencies, expressed as a percentage, experienced during the Platform Preparation reduction behaviours.

	**Pressure range (kPa)**	**Sensor (%)**											
		**DP1**	**PP1**	**DP2**	**IP2**	**PP2**	**DP3**	**IP3**	**PP3**	**DP4**	**PP4**	**DP5**	**PP5**
**LAH [illegible]**	**0-20**	2.6	23.9	7.9	16.4	9.2	27.0	24.1	46.2	24.0	34.7	8.0	37.9
	**20.1 - 40**	21.6	48.4	32.2	32.2	35.8	56.9	39.5	43.1	51.9	47.1	37.0	55.7
	**40.1 - 60**	28.4	17.4	30.6	22.8	34.1	8.8	18.5	7.7	13.5	11.6	31.5	6.4

	**60.1 - 80**	20.1	8.4	14.4	16.4	11.8	4.4	11.1	1.5	8.7	5.0	13.9	0
	**80.1 - 100**	12.4	1.3	8.3	6.4	6.1	2.2	3.1	1.5	1.9	0.8	5.0	0
	**100.1 - 120**	7.7	0	2.1	4.1	0.4	0	2.5	0	0	0.8	2.9	0
	**120.1 - 140**	2.1	0.6	2.5	1.2	2.2	0	0.6	0	0	0	1.3	0
	**140.1 - 160**	2.6	0	1.2	0.6	0.4	0	0	0	0	0	0.4	0
	**160.1 - 180**	1.0	0	0.4	0	0	0	0	0	0	0	0	0
	**180.1 - 200**	1.5	0	0	0	0	0	0.6	0	0	0	0	0
	**200.1 - 220**	0	0	0	0	0	0	0	0	0	0	0	0
	**220.1 - 240**	0	0	0.4	0	0	0.7	0	0	0	0	0	0
	**240.1 - 260**	0	0	0	0	0	0	0	0	0	0	0	0

Supplementary Table 10: Descriptive data for the twelve sensors during the four types of stone tool reduction, without the inclusion of participant seven. 'DP', 'IP' and 'PP' correspond to the distal phalanx, intimidate phalanx and proximal phalanx sensors, respectively. Sensors numbers correspond to the respective digit. Minimum values always equal 15 kPa, except for DP1 during the Oldowan reduction, which recorded a minimum value of 17.5 kPa. These data exclude values of '0' when sensors were not loaded.

		**Sensor**											
		**DP1**	**PP1**	**DP2**	**IP2**	**PP2**	**DP3**	**IP3**	**PP3**	**DP4**	**PP4**	**DP5**	**PP5**
**Oldowan Flake**	**Mean**	61.6	34.6	40.2	28.6	40.8	26.4	47.9	40.7	30.7	26.4	40.4	28.5
	**SD**	29.3	19.3	20.9	13.3	22.1	13.9	25.4	30.3	19.1	11.0	20.	14.7
	**Max**	162.5	112.5	125	85	155	85	157.5	195	100	77.5	120	90
**Early Acheulean Handaxe**	**Mean**	54.9	31.8	39.3	25.4	41.0	34.6	40.1	34.6	35	27.4	40.6	27.8
	**SD**	26.3	16.6	19.8	11.7	24.4	27.2	22.6	25.8	21.2	12.4	21.9	12.6
	**Max**	172.5	95	145	75	252.5	162.5	192.5	152.5	110	87.5	162.5	75
**Late Acheulean Handaxe**	**Mean**	50.0	32.3	47.4	42.0	39.3	32.1	37.1	27.3	32.3	28.2	46.0	28.6
	**SD**	27.3	20.9	29.6	26.8	25.3	15.5	24.8	14.6	18.6	14.4	24.0	11.6
	**Max**	190	162.5	227.5	207.5	232.5	82.5	197.5	115	132.5	132.5	155	92.5
**LAH Platform Preparation**	**Mean**	67.5	34.1	53.4	48.8	47.0	33.9	38.3	25.0	34.0	31.0	47.2	25.4
	**SD**	34.7	18.7	31.6	27.7	24.2	22.9	26.0	13.5	18.1	17.5	24.8	8.7
	**Max**	187.5	130	232.5	147.5	142.5	222.5	197.5	87.5	97.5	115	145	57.5

Supplementary Table 11: Digit recruitment frequencies expresses as a percentage relative the total number of manual behaviour recorded (without participant seven's data).

		**Sensor**											
		**PD1**	**PP1**	**PD2**	**IP2**	**PP2**	**PD3**	**IP3**	**PP3**	**PD4**	**PP4**	**PD5**	**PP5**
Oldowan Flake (n = 482)	**Instances**	377	143	310	146	306	91	305	99	106	64	297	121
	**Frequency (%)**	78	29.7	64.3	30.3	63.5	18.9	63.3	20.5	22.0	13.3	61.6	25.1
EAH (n = 723)	**Instances**	499	181	484	228	492	155	459	164	140	123	523	200
	**Frequency (%)**	69	25.0	66.9	31.5	68.0	21.4	63.5	22.7	19.4	17.0	72.3	27.7
LAH (n = 1209)	**Instances**	761	417	878	638	877	424	636	545	400	458	918	609
	**Frequency (%)**	63	34.5	72.6	52.8	72.5	35.1	52.6	45.1	33.1	37.9	75.9	50.4
PP (n = 258)	**Instances**	194	146	233	161	216	132	152	118	101	115	226	130
	**Frequency (%)**	75.2	56.6	90.3	62.4	83.7	51.2	58.9	45.7	39.1	44.6	87.6	50.4

Supplementary Table 12: Pressure magnitude frequencies, expressed as a percentage, experienced during the Oldowan reduction sequence (minus participant seven).

	Pressure range (kPa)	Sensor (%)											
		DP1	PP1	DP2	IP2	PP2	DP3	IP3	PP3	DP4	PP4	DP5	PP5
Oldowan Flake	0-20	1.6	23.1	19.4	34.9	11.8	56.0	12.5	27.3	34.9	34.4	15.2	39.7
	20.1 - 40	29.2	51.0	41.3	47.9	50.3	31.9	31.1	36.4	50.9	56.2	44.1	44.6
	40.1 - 60	27.3	18.2	22.3	15.1	23.5	9.9	36.1	24.2	5.7	7.8	25.6	11.6
	60.1 - 80	20.7	4.2	12.3	1.4	8.5	1.1	12.1	6.1	2.8	1.6	12.5	2.5
	80.1 - 100	10.1	1.4	3.5	0.7	3.6	1.1	3.9	2.0	5.7	0	2.0	1.7
	100.1 - 120	4.8	2.1	1.0	0	1.3	0	2.0	1.0	0	0	0.7	0
	120.1 - 140	5.0	0	0.3	0	0.7	0	1.3	0	0	0	0	0
	140.1 - 160	1.1	0	0	0	0.3	0	1.0	2.0	0	0	0	0
	160.1 - 180	0.3	0	0	0	0	0	0	0	0	0	0	0
	180.1 - 200	0	0	0	0	0	0	0	1.0	0	0	0	0
	200.1 - 220	0	0	0	0	0	0	0	0	0	0	0	0
	220.1 - 240	0	0	0	0	0	0	0	0	0	0	0	0
	240.1 - 260	0	0	0	0	0	0	0	0	0	0	0	0

Supplementary Table 13: Pressure magnitude frequencies, expressed as a percentage, experienced during the Early Acheulean Handaxe reduction sequence (minus participant seven).

	Pressure range (kPa)	Sensor (%)											
		DP1	PP1	DP2	IP2	PP2	DP3	IP3	PP3	DP4	PP4	DP5	PP5
Early Acheulean Handaxe	0-20	1.6	32.0	13.6	42.5	16.3	40.0	18.7	40.2	26.4	30.9	16.8	36.5
	20.1 - 40	36.7	40.3	50.0	49.1	43.1	36.1	44.4	32.3	45.7	61.0	42.1	49.5
	40.1 - 60	30.7	21.0	25.6	4.8	23.8	12.3	20.9	15.9	17.1	4.1	27.2	11.5
	60.1 - 80	15.4	5.0	7.0	3.5	10.8	4.5	11.1	5.5	5.7	3.3	8.2	2.5
	80.1 - 100	9.0	1.7	1.7	0	3.3	2.6	3.5	2.4	2.9	0.8	4.0	0
	100.1 - 120	4.0	0	1.7	0	2.2	1.3	0.7	1.2	2.1	0	1.3	0
	120.1 - 140	1.6	0	0.2	0	0.4	1.9	0	1.8	0	0	0.2	0
	140.1 - 160	0.8	0	0.2	0	0	0.6	0.4	0.6	0	0	0	0
	160.1 - 180	0.2	0	0	0	0	0.6	0	0	0	0	0.2	0
	180.1 - 200	0	0	0	0	0	0	0.2	0	0	0	0	0
	200.1 - 220	0	0	0	0	0	0	0	0	0	0	0	0
	220.1 - 240	0	0	0	0	0	0	0	0	0	0	0	0
	240.1 - 260	0	0	0	0	0.2	0	0	0	0	0	0	0

Supplementary Table 14: Pressure magnitude frequencies, expressed as a percentage, experienced during the Late Acheulean Handaxe reduction sequence (minus participant seven).

	Pressure range (kPa)	Sensor (%)											
		DP1	PP1	DP2	IP2	PP2	DP3	IP3	PP3	DP4	PP4	DP5	PP5
Late Acheulean Handaxe	0-20	5.5	32.6	11.5	15.8	21.8	29.2	26.1	40.0	26.3	38.0	10.2	28.1
	20.1 - 40	41.3	47.0	42.1	48.3	42.1	49.5	43.6	48.3	52.3	48.7	40.0	60.3
	40.1 - 60	28.0	11.3	21.6	18.5	22.6	13.0	19.8	7.9	14.3	9.8	29.6	9.4
	60.1 - 80	13.8	5.5	12.4	8.5	7.0	7.8	5.5	2.2	4.0	2.8	10.5	2.1
	80.1 - 100	5.5	1.9	7.2	3.9	4.0	0.5	2.4	1.3	1.8	0.2	6.2	0.2
	100.1 - 120	3.7	1.0	2.1	3.0	1.5	0	0.9	0.4	1.0	0.2	2.3	0
	120.1 - 140	0.9	0	1.5	1.1	0.2	0	0.3	0	0.5	0.2	1.0	0
	140.1 - 160	0.9	0.5	0.7	0.8	0.3	0	0.8	0	0	0	0.2	0
	160.1 - 180	0.3	0.2	0.5	0	0.3	0	0.5	0	0	0	0	0
	180.1 - 200	0.1	0	0.3	0	0	0	0.2	0	0	0	0	0

	**200.1 - 220**	0	0	0.1	0.2	0.1	0	0	0	0	0	0	0
	**220.1 - 240**	0	0	0	0	0.1	0	0	0	0	0	0	0
	**240.1 - 260**	0	0	0	0	0	0	0	0	0	0	0	0

Supplementary Table 15: Pressure magnitude frequencies, expressed as a percentage, experienced during the Platform Preparation reduction behaviours (minus participant seven).

	**Pressure range (kPa)**	**Sensor (%)**											
		**DP1**	**PP1**	**DP2**	**IP2**	**PP2**	**DP3**	**IP3**	**PP3**	**DP4**	**PP4**	**DP5**	**PP5**
**LAH Platform Preparation**	**0-20**	2.6	23.3	7.7	17.4	9.3	28.0	25.7	50	22.8	34.8	8.4	38.5
	**20.1 - 40**	21.6	50.0	33.0	28.6	37.5	56.1	40.1	41.5	52.5	47.0	37.2	56.2
	**40.1 - 60**	28.4	16.4	29.2	24.2	31.9	8.3	18.4	5.1	13.9	11.3	32.3	5.4
	**60.1 - 80**	20.1	8.2	14.6	16.8	12.5	4.5	10.5	1.7	2.0	5.2	12.8	0
	**80.1 - 100**	12.4	1.4	8.6	6.8	6.0	2.3	2.6	1.7	0	0.9	4.9	0
	**100.1 - 120**	7.7	0	2.1	4.3	0	0	2.0	0	0	0.9	2.7	0
	**120.1 - 140**	2.1	0.7	2.6	1.2	2.3	0	0	0	0	0	1.3	0
	**140.1 - 160**	2.6	0	1.3	0.6	0.5	0	0	0	0	0	0.4	0
	**160.1 - 180**	1.0	0	0.4	0	0	0	0	0	0	0	0	0
	**180.1 - 200**	1.5	0	0	0	0	0	0.7	0	0	0	0	0
	**200.1 - 220**	0	0	0	0	0	0.8	0	0	0	0	0	0
	**220.1 - 240**	0	0	0.4	0	0	0	0	0	0	0	0	0
	**240.1 - 260**	0	0	0	0	0	0	0	0	0	0	0	0
